# Reproductive Health Experiences of Females Diagnosed with Young-Onset Colorectal Cancer: A Multi-Method Cross-Sectional Survey

**DOI:** 10.3390/curroncol29020042

**Published:** 2022-01-21

**Authors:** Louise Araujo, Nevena Rebic, Hallie Dau, Lori Brotto, Manju George, Mary A. De Vera

**Affiliations:** 1 Faculty of Pharmaceutical Sciences, University of British Columbia, 2405 Wesbrook Mall, Vancouver, BC V6T 1Z3, Canada; laraujo7@student.ubc.ca (L.A.); nevena.rebic@ubc.ca (N.R.); hallie.dau@ubc.ca (H.D.); 2 Collaboration for Outcomes Research and Evaluation, Vancouver, BC V6T 1Z3, Canada; 3 Department of Obstetrics and Gynaecology, Faculty of Medicine, University of British Columbia, Vancouver, BC V6T 1Z3, Canada; Lori.Brotto@vch.ca; 4 COLONTOWN^®^, Paltown Development Foundation 969 Diggs Road, Crownsville, MD 21032, USA; manjuggm@gmail.com

**Keywords:** colorectal cancer, reproductive health, sexual health, pregnancy

## Abstract

Objective: Given the increasing risk of young-onset colorectal cancer (yCRC) among adults under 50 years, it is important to understand impacts on reproductive health. Our objective was to assess experiences with reproductive health after yCRC diagnosis among females. Methods: We conducted a cross-sectional study among females, 18 years or older, who have been diagnosed yCRC and are able to communicate in English. Data were gathered using an online survey involving both quantitative (e.g., multiple choice) and qualitative (e.g., open-ended text) questions on pregnancy history, influence of yCRC on reproductive decisions, and experiences with reproductive healthcare. Results: Altogether, 101 females with yCRC participated, including 23 who had never been pregnant and 78 who had been pregnant. yCRC influenced family planning goals for one-third of participants. Furthermore, compared to participants who completed treatment, those currently undergoing treatment had higher odds of indicating their yCRC diagnosis influenced family planning goals (adjusted odds ratio 4.93; 95% confidence interval 1.29 to 18.78). Although 53 (52.5%) participants indicated having discussions regarding reproductive health with healthcare provider(s), 44 (43.6%) did not. Content analysis of open-ended survey questions identified themes on the emotional impacts, experiences with reproductive healthcare, reproductive and family planning considerations, and the related issue of sexual health impacts of yCRC. Conclusions: Gaps in care, related to limited reproductive health discussions, influence of yCRC on family planning, and experiencing lasting reproductive health impacts highlight the need for improving reproductive healthcare, particularly for females diagnosed with yCRC.

## 1. Introduction

Reproductive years among females range from 15 to 49 years of age [1] and in the past 50 years, the average age for a first pregnancy has increased from 24 years of age in the 1970’s [2] to 29.2 years of age in 2016 [3]. Since the likelihood of cancer increases with age, females today are more likely to be diagnosed with cancer during their reproductive years. Although the incidence of cancer during pregnancy is low (0.07–0.1%), cancer is a leading cause of death among females of childbearing age (15–34 years of age) [4,5].

Colorectal cancer (CRC) has historically been considered a disease of older persons. However, growing evidence on the increasing incidence of young-onset CRC (yCRC) in individuals below 50 years of age [6,7,8,9,10] including a 2020 worldwide systematic review that estimated a pooled annual percent change in incidence (APCi) for yCRC of 1.33% (95% confidence interval (95% CI), 0.97 to 1.68) [11]. Given the increasing risk of yCRC and its estimated occurrence in two out of every 100,000 pregnancies [12], it is important to consider implications among younger adults, particularly impacts on reproductive health, which may span fertility, pregnancy, and early menopause.

Prior research on yCRC and reproductive health is limited and has largely focused on clinical aspects during pregnancy and difficulties of diagnosing yCRC given that symptoms tend to be similar to those experienced in normal pregnancies (e.g., nausea, vomiting, constipation, and anemia) [4,13,14]. Understanding of reproductive health impacts, particularly on family planning decisions, of yCRC among women who experienced pregnancy around the time of their yCRC diagnosis (e.g., before or after) as well as those who have never been pregnant are lacking. In general, cancer patients who are unaware of post-treatment fertility options experience more conflict regarding future family planning decisions [15]. There also tends to be discrepancies between what patients and healthcare providers find to be helpful when discussing reproductive health [16]. These discrepancies between patient and healthcare provider expectations around reproductive health can impact the care received [17]. As these prior studies have not been conducted among those with yCRC, it remains unclear whether women are having discussions about the reproductive health implications of yCRC with their healthcare providers. To address these gaps, our objectives were to: (1) characterize reproductive health outcomes of women after yCRC diagnosis according to pregnancy history (e.g., never been pregnant, have been pregnant); (2) determine how yCRC diagnosis influences family planning decisions; and (3) assess experiences with reproductive healthcare, including discussions with healthcare provider(s).

## 2. Methods

### 2.1. Participant Recruitment

We invited females who are 18 years or older, have received a diagnosis of yCRC (before the age of 50 years), and are able to communicate in English. To recruit participants, we used the authors’ and their affiliated institutions’ social media channels (e.g., Twitter, Facebook, and Instagram) as well as drawing from a list of individuals who had participated in previous CRC-related research conducted by our research team and indicated their consent to be notified of future studies [18,19,20,21]. Individuals were directed to a study website, designed using Qualtrics, an online survey platform, which included information about the objective of the study, eligibility criteria, and what participation involves. Individuals who consented to participate were directed to the study survey; individuals who do not wish to participate were asked to close their browser.

### 2.2. Study Design and Data Collection

We conducted a cross-sectional study and administered an online health survey which included 12 pages and consisted of four sections (Supplementary Figure S1). The first section on yCRC characteristics comprised nine quantitative questions, with multiple choice or drop-down response formats: on type of cancer (e.g., ‘colon’, ‘rectal’, ‘both sites’), age at diagnosis, date of diagnosis, stage, symptoms (e.g., ‘blood in the stool’, ‘diarrhea’, ‘constipation’), treatments received (e.g., ‘radiation’, ‘surgery’, ‘chemotherapy’), and treatment status (‘completed’ or ‘in treatment’). The second section covered questions on pregnancy history (e.g., ‘never been pregnant,’ ‘have been pregnant’), when the pregnancy occurred in relation to yCRC diagnosis (e.g., ‘before’, ‘after’), estimated start of pregnancy, and whether pregnancy resulted in a live birth. This section also included questions on reproductive decisions, namely on family planning, such as whether yCRC diagnosis influenced future reproductive decisions (e.g., ‘yes’, ‘no’), whether they considered having children after yCRC diagnosis, and, if so, family building options considered (e.g., ‘childbearing’, ‘adoption’, ‘surrogacy’, ‘assisted reproductive technology’). An open-ended question provided participants the opportunity to indicate how their yCRC influenced or changed their reproductive decisions. The third section included seven questions on reproductive healthcare in relation to their yCRC diagnosis. Questions included whether they received discussion regarding reproductive health during treatment for yCRC (e.g., ‘yes’, ‘no’), who initiated the discussion (e.g., ‘myself’, ‘healthcare provider’), and the type(s) of healthcare provider(s) involved in the discussion (e.g., ‘oncologist’, ‘surgeon’). This section also included questions on reproductive health topics covered (e.g., ‘hormone replacement’, ‘menopause’) and resources provided (e.g., ‘pamphlets’). The final question in this section was open-ended and invited participants to share, in a textbox, anything else they wished they were told about reproductive health and yCRC. The fourth section on demographic information consisted of six questions, including country of residence, current age, marital status, level of education, ethnicity, and area of living (e.g., ‘urban’, ‘rural’).

### 2.3. Statistical Analysis

We analyzed close-ended quantitative questions by calculating descriptive statistics to characterize participants and summarize their responses. Given potential differences in experiences according to history of pregnancy, we grouped participants accordingly (e.g., ‘never been pregnant’, ‘have been pregnant’) and compared characteristics and outcomes using Chi-square tests. In exploratory analyses, we evaluated the impact of yCRC diagnosis on reproductive decisions by creating a binary categorical outcome representing participants’ responses on whether their yCRC diagnosis influenced family planning decisions (‘yes’, ‘no’) and evaluated determinants using multiple logistic regression. Potential determinants represent variables we surveyed on such as: age at yCRC diagnosis, treatment status (in treatment versus completed treatment), and education (less than college versus college or more). To support quantitative analyses, we used Microsoft Excel 2016, Qualtrics XM Stats iQ, and SPSS.

### 2.4. Qualitative Analysis

We exported all narrative responses to open-ended qualitative questions into NVivo 12 (QSR International), which was used to categorize our analysis. We applied descriptive content analysis [22], following three coding steps of initial open coding (assigning concepts to phrases and sections of the text responses), sorting and organization into categories, and construction into themes. Initial coding was conducted by the first author (LA). Subsequently, three study authors (LA, NR, and MDV) were involved in the formation of categories and the themes and collaborated to reach consensus for the final reporting.

### 2.5. Ethical Approval and Consent

We obtained ethical approval from the University of British Columbia Behavioural Research Ethics Board. Data were collected and stored securely using an online survey platform, a survey platform that is compliant with the British Columbia (BC) Freedom of Information and Protection of Privacy Act and meets institutional and jurisdictional privacy requirements. Participants provided informed consent prior to participating in the survey and their confidentiality was maintained throughout the study.

## 3. Results

Between 20 March 2020 and 22 April 2020, 121 individuals accessed the survey. We excluded 20 records with incomplete responses. Among 101 participants included, 23 (22.8%) had never been pregnant and 78 (77.2%) had been pregnant at least once (Table 1). As shown in Table 2, characteristics of yCRC were similar between groups according to pregnancy history. For women who had never been pregnant, colon and rectal cancer were equally diagnosed (11, 47.8% for both) and the most commonly reported symptoms were blood in the stool (15, 65.2%), gas/cramps/feeling bloated (10, 43.5%), narrow stool (9, 39.1%), and weakness/fatigue (9, 39.1%). For women who had been pregnant, 47 (60.3%) were diagnosed with colon cancer with the most commonly reported symptoms being blood in the stool (53, 67.9%), gas/cramps/feeling bloated (39, 50%), and narrow stool (33, 42.3%). Of note, we observed a higher proportion of women who had never been pregnant report bowel obstruction as a symptom as compared to those who had been pregnant (34.8% vs. 11.5%, Chi-square *p*-value = 0.009).

### 3.1. Experiences with Reproductive Health after yCRC Diagnosis—Quantitative Responses

**Figure 1:**
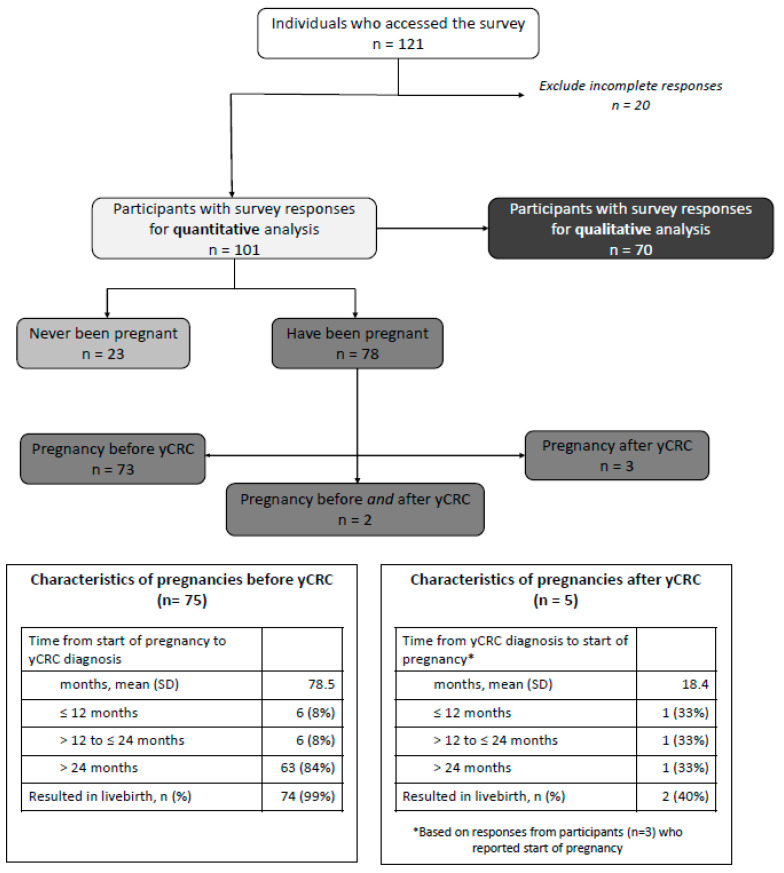
Participant flow and characteristics according to pregnancy history.

As shown in Figure 1, among participants that had been pregnant, 73 (93.6%) were pregnant before yCRC diagnosis, three (3.8%) were pregnant after yCRC diagnosis, and two (2.6%) experienced pregnancies before and after yCRC diagnosis. Of the 75 pregnancies that occurred before yCRC diagnosis, 74 (98.7%) resulted in live births. With respect to timing of yCRC diagnosis and pregnancy, six participants had their diagnosis within 12 months, six within 12–24 months, and 63 greater than 24 months from the start of pregnancy. Of the five pregnancies that occurred after yCRC diagnosis, two (40%) resulted in a live birth. With respect to timing of yCRC diagnosis and start date of pregnancy, one participant became pregnant within 12 months of their yCRC diagnosis, one within 24 months, and one at greater than 24 months.

With respect to the impacts of yCRC on reproductive decisions, one-third of participants (34, 33.7%) indicated that their yCRC diagnosis influenced their family planning goals, including nine (39.1%) of those who had never been pregnant and 25 (32.1%) of those who had been pregnant. As shown in Table 3, age at yCRC diagnosis was a predictor, particularly among participants diagnosed at between 20 and 29 years (adjusted odds ratio (aOR), 22.73; 95% confidence interval (CI), 3.53 to 146.39) and 30 to 39 years (aOR, 21.94; 95% CI, 5.59 to 86.18) as compared to those diagnosed at between 40 and 49 years. Participants currently undergoing treatment were more likely to indicate that their yCRC diagnosis influenced their reproductive decisions as compared to those who had completed treatment (aOR, 4.93; 95% CI, 1.29 to 18.78). When asked whether they had considered having children *after* their yCRC diagnosis, one-fifth (20, 19.8%) of participants indicated that this remains of interest, including options of childbearing, surrogacy, and adoption. Of note, we found that more participants who had never been pregnant than those who had been pregnant indicated that they were considering surrogacy (83.3% versus 28.6%, Chi-square *p*-value = 0.01) and adoption (50.0% versus 14.3%, Chi-square *p*-value = 0.04).

**Figure 2:**
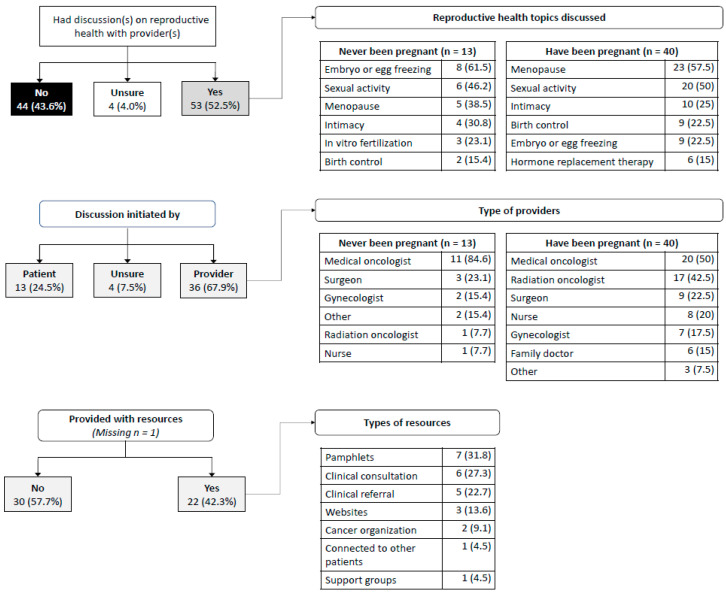
Experiences with having discussion(s) about reproductive health with healthcare providers.

With respect to having discussions regarding reproductive health, 53 (52.5%) participants indicated having discussions with healthcare provider(s) after being diagnosed with yCRC, 44 (43.6%) did not, and 2 (4.0%) were unsure if these discussions occurred (Figure 2). Among those that had discussions, the majority (36, 67.9%) indicated that they were initiated by healthcare provider(s) and nearly a quarter (24.5%) of participants indicated they were initiated by themselves. The most frequently discussed topics among participants who had never been pregnant was embryo/egg freezing (61.5%) followed by sexual activity (46.2%) and menopause (38.5%). Among participants who had been pregnant, the most frequently discussed topics were menopause (57.5%), sexual activity (50%), and intimacy (25%). The healthcare providers that most frequently discussed reproductive health after diagnosis were medical oncologists (84.6% among participants who had never been pregnant; 50% among those who had been pregnant). Among participants who had discussions regarding reproductive health, 57.7% indicated that they were not provided with any additional resources (e.g., brochures or websites).

### 3.2. Experiences with Reproductive Health after yCRC Diagnosis—Qualitative Responses

Finally, 70 participants provided narrative responses to open-ended questions regarding reproductive health experiences in relation to their yCRC diagnosis. The average response length was 44 ± 11 words. Descriptive content analyses identified four themes. The first theme on the emotional impacts of yCRC reflects how the psychological and reproductive burden of a cancer diagnosis are closely related. This theme includes three categories of: (1) processing yCRC diagnosis; (2) worries and fears; and (3) coping strategies. The second theme, experiences with reproductive healthcare after yCRC diagnosis, encompassed whether participants had a discussion with healthcare providers or not and the resources they were offered. We noted a range of experiences including from those who (1) did not have a discussion with healthcare providers (“no discussion was ever had”) as well those who (2) received a referral for reproductive healthcare or (3) had a discussion with healthcare providers. A category within this theme, (4) online resources, also captured experiences with information and support gained from these sources (“found online forums a huge help in this area”). The third theme, reproductive and family planning considerations with yCRC diagnosis, touched on reasons women chose not to have children after diagnosis and the impact yCRC diagnosis had on family planning. This theme consisted of four categories: (1) reproductive and pregnancy history; (2) impact of yCRC on family planning; (3) role of genetic testing (“my health has pushed me towards not having children”); and (4) fertility. The fourth theme revealed a related aspect of sexual health impacts of yCRC capturing areas where yCRC and treatments had devastated participants, specifically, (1) vaginal side effects (“damage to vagina”) and (2) intimacy and intercourse (“difficult sex”). Impacts tended to be issues that participants did not anticipate and were not discussed with healthcare providers prior to participants experiencing them. Table 4 provides a summary along with representative quotes.

## 4. Discussion

In this cross-sectional study, we characterized the reproductive health experiences of 101 women who were diagnosed with yCRC, including reproductive decisions and the reproductive healthcare received, taking into consideration pregnancy history where relevant. Indeed, a diagnosis of yCRC is associated with considerable reproductive health burden with one-third of participants (34, 33.7%) indicating that their diagnosis influenced their family planning goals. Although just over half (52.5%) of participants indicated having discussions regarding reproductive health with healthcare provider(s), 43.6% did not and a further 4.0% were unsure. Providing further context to quantitative responses, qualitative responses highlighted care and information gaps (*“I wish a longer discussion and basic overview of my reproductive health was done”*).

Evidence in recent years on the increasing risk of yCRC has mobilized efforts into not only understanding and addressing this risk but also identifying areas to support patients and improve both short- and long-term outcomes [23,24,25,26]. Indeed, treatments for yCRC, including pelvic surgery, radiotherapy, as well as chemotherapy, have detrimental effects on reproductive health outcomes [27], necessitating investigations on impacts among patients. Survey questions regarding having pregnancies and corresponding timing related to yCRC diagnosis allowed us to characterize these experiences, which had not been achieved in prior studies. Indeed, with an estimated incidence of CRC during 2 of every 100,000 pregnancies [12], yCRC is among the cancers with negative implications on pregnancy. Even so, prior research on yCRC and pregnancy is limited and has largely focused on clinical aspects during pregnancy such as treatment options, maternal and fetal outcomes, and effects of treatment on reproduction [12,28] as well as difficulties of diagnosing CRC during pregnancy given the similarity to symptoms experienced in normal pregnancy (e.g., nausea, vomiting, constipation, and anemia) [4,13,14]. A study on specific and non-specific symptoms of CRC by Rasmussen et al. found that women of 20–39 years of age experienced the following symptoms in association with CRC: abdominal pain (38.0%), blood in stool (7.7%), diarrhea (19.9%), constipation (23.5%), and tiredness (73.4%) [29]. We were also interested in surveying participant symptoms as common symptoms of yCRC are also typically experienced post-partum. Aside from confirming prior findings by Rasmussen et al. particularly among participants who had been pregnant, our study provides new insights on symptoms experienced by women diagnosed with yCRC who had never been pregnant. Particularly, we observed that bowel obstruction was reported at a higher frequency among those who had never been pregnant compared to those who had been pregnant (34.8% vs. 11.5%; Chi-square *p*-value = 0.009). Nonetheless, we did not observe any other differences between participants who had never been pregnant and who had been pregnant with respect to yCRC characteristics (CRC type, stage) or treatments.

A concern highlighted in our study was the impact of yCRC on reproductive decisions, with a third of participants indicating that their yCRC diagnosis influenced their family planning goals, including 39.1% who had never been pregnant and 32.1% who had been pregnant. For many participants, this meant a change from wanting children to deciding not to have children. Drawing insights from qualitative analyses of open-ended survey responses allowed us to identify reasons participants chose not to have children after yCRC diagnosis. Aside from the direct impacts of yCRC and treatments (“After so much surgery and invasion of my body the thought of giving birth or being pregnant makes me feel dread rather than any sense of excitement”), genetic testing was a factor for changing their family goals for 11.4% of participants. Concerns about recurrence due to genetic predisposition prompted some participants to choose not to have children. For others, the fear of passing on genetic predisposition for CRC determined decisions not to have children.

Another concern identified in our study is a large proportion of participants, 43.6%, indicating that they did not engage in a discussion about reproductive health while undergoing treatment for yCRC. Our qualitative analyses of responses to open-ended questions provide insights into these findings, as they were designed to allow participants to indicate what topics are most important to them and bring to light issues and concerns that they may have such as impacts of CRC treatments on fertility and resultant early menopause. Qualitative analyses also revealed the importance of the related issue of sexual health impacts of yCRC with participants sharing issues they did not anticipate or were often not discussed with healthcare providers, namely vaginal side effects, intimacy, and intercourse (mentioned in 11.4%, 7.1%, and 7.1% of text responses, respectively). These concerns, as described by Schover in a 2007 report on reproductive issues after cancer, are common concerns for patients of young onset cancers [30] and are now demonstrated in our current study for women with yCRC. It is important to note that previous studies on patient–healthcare provider discussions on reproductive health have, in general, found discrepancies between expectations. In their 2016 study of 346 young adult female cancer survivors including 27 with yCRC, Benedict et al. found that 35% of study participants did not believe they had enough support to make reproductive decisions [15]. They also found that conflict around decisions was associated with having higher unmet fertility information. Lack of support for cancer patients’ informational needs likely comes from a disconnect between patients and healthcare providers on what information is useful. Canzona et al. found that between patients and healthcare providers, there was a divide in what constituted helpful communication with patients, indicating that they preferred direct recommendations, verbally acknowledged distress, and not being questioned on the importance of their concerns [16].

The strengths and limitations of our study warrant discussion. A strength of our study was the multi-method approach whereby quantitative findings were supported and/or further contextualized by qualitative findings. However, as our study was administered as an online survey, this may have prevented individuals with limited access to the internet from participating. By allowing participants to choose whether to complete the survey or not, we do not have any knowledge about individuals that chose not to access the survey or to end the survey before completing. Although all 101 participants provided responses to the quantitative questions in the survey, 31 did not provide responses to the qualitative questions and we were not able to gather information on reasons for these non-responses. In addition, by allowing self-reporting through a survey, there may be bias and reduced accuracy to the participants’ responses. On the other hand, given the sensitivity of the topic (i.e., reproductive and sexual health and family planning), it is likely that this method of gathering confidential information provided the needed privacy to allow participants to share their experiences in a way that would have been attenuated with face-to-face data collection. Among participants who had been pregnant, the majority occurred >24 months before yCRC diagnosis. As well, the distribution of participants’ current age suggest that many have received yCRC diagnosis for more than 10 years. Taken together, there may be potential limitations with participants’ recall. We also advise that caution be taken in the interpretation of some of the quantitative findings, particularly with respect to influence of age at yCRC diagnosis on family planning given that the study sample size may have resulted in imprecise estimates. Finally, although our sample consisted of predominantly white participants (90.9% of those ever having been pregnant; 80.5% of those having been pregnant) with a postsecondary education or higher (100% of those never having been pregnant; 93.6% of those having been pregnant) and we limited inclusion to English-speaking participants, we identified important gaps in their reproductive and sexual health care. It is critical that further studies examine the experience of patients with historically marginalized racial, gender, and sexual identities.

## 5. Conclusions

In conclusion, through a multi-method approach our findings provide a better understanding of the reproductive health experiences of women diagnosed with yCRC. The influence of yCRC on future family planning decisions along with gaps in care, particularly related to limited reproductive care discussions for a number of participants, highlight the need for improving the reproductive health standard of care for women with yCRC.

## Figures and Tables

**Table 1:** Participant demographic characteristics according to pregnancy history.

Characteristic *n* (%)	Never Been Pregnant (*n* = 23)	Have Been Pregnant (*n* = 78)	*p*-Value ^a^
Current age (years)			
20–29	1 (4.3)	2 (2.6)	0.51
30–39	9 (39.1)	16 (20.5)	
40–49	8 (34.8)	39 (50.0)	
50–59	4 (17.4)	18 (23.1)	
60–69	1 (4.3)	2 (2.6)	
70–79	0	1 (1.3)	
Country			
Canada	8 (34.8)	37 (48.7)	0.15
USA	10 (43.5)	32 (42.1)	
UK	1 (4.3)	4 (5.3)	
Other ^b^	4 (5.1)	3 (3.9)	
Ethnicity			
White	20 (90.9)	62 (80.5)	0.66
Hispanic	0	2 (2.6)	
Black	0	2 (2.6)	
Asian	0	6 (7.8)	
Native/Aboriginal	0	1 (1.3)	
Middle Eastern	0	1 (1.3)	
Other ^c^	2 (9.1)	3 (3.9)	
Residence			
Urban	14 (60.9)	21 (26.9)	0.03
Suburban	6 (26.1)	41 (52.6)	
Rural	3 (13.0)	15 (19.2)	
Do not know	0	1 (1.3)	
Education level			
High school or less	0	5 (6.4)	0.21
Postsecondary or more	23 (100)	73 (93.6)	
Marital status			
Married/common-law	17 (73.9)	67 (85.9)	<0.0001
Single, never married	6 (26.1)	1 (1.3)	
Separated/divorced/widowed	0	10 (12.8)	

^a^ Calculated using Chi-square test. ^b^ Australia, France, Ireland, Italy, Sweden. ^c^ Includes respondents that indicated >1 ethnicity.

**Table 2:** CRC characteristics according to pregnancy history.

CRC Characteristic *n* (%)	Never Been Pregnant (*n* = 23)	Have Been Pregnant (*n* = 78)	*p*-Value ^a^
Age at Diagnosis			
20–29	3 (13.0)	6 (7.8)	0.50
30–39	11 (47.8)	28 (36.4)	
40–49	9 (39.1)	43 (55.8)	
CRC Type			
Colon	11 (47.8)	47 (60.3)	0.52
Rectal	11 (47.8)	27 (34.6)	
Both Sites	1 (4.3)	4 (5.1)	
CRC Stage			
Stage 0	0	3 (3.8)	0.67
Stage I	1 (4.3)	3 (3.8)	
Stage II	6 (26.1)	10 (12.8)	
Stage III	11 (47.8)	41 (52.6)	
Stage IV	4 (17.4)	18 (23.1)	
Do not know	1 (4.3)	3 (3.8)	
Treatment Status			
In treatment	7 (30%)	17 (22%)	0.41
Completed treatment	16 (70%)	61 (78%)	
Number of Treatment Modalities			
More than one	18 (78.3)	72 (92.3)	0.16
One	4 (17.4)	5 (6.4)	
None	1 (4.3)	1 (1.3)	
Treatment Type ^b,c^			
Chemotherapy	21 (91.3)	70 (89.7)	0.83
Surgery	19 (82.6)	72 (92.3)	0.17
Radiation	7 (30.4)	35 (44.9)	0.22
Immunotherapy	0	7 (9.0)	0.14
None	1 (4.3)	1 (1.3)	0.35
Symptoms experienced ^b,c^			
Blood in the stool	15 (65.2)	53 (67.9)	0.81
Gas, cramps, feeling bloated	10 (43.5)	39 (50.0)	0.58
Narrow stool	9 (39.1)	33 (42.3)	0.79
Weakness or fatigue	9 (39.1)	30 (38.5)	0.95
Constipation	8 (34.8)	19 (24.4)	0.32
Bowel obstruction	8 (34.8)	9 (11.5)	0.009
Diarrhea	7 (30.4)	24 (30.8)	0.98
Weight loss	7 (30.4)	20 (25.6)	0.65
Pain in the abdomen, back, buttocks, or legs	7 (30.4)	16 (20.5)	0.32
Anemia	6 (26.1)	31 (39.7)	0.23
Nausea or vomiting	6 (26.1)	11 (14.1)	0.18
Loss of appetite	5 (21.7)	11 (14.1)	0.38
Bleeding from the rectum	4 (17.4)	21 (26.9)	0.35
Rectal pain or discomfort	4 (17.4)	15 (19.2)	0.84
Other	1 (4.3)	9 (11.5)	0.31
Lump in the abdomen or rectum	1 (4.3)	6 (8.0)	0.58
Fluid buildup in the abdomen	1 (4.3)	4 (5.1)	0.88
Swollen lymph nodes	1 (4.3)	2 (2.6)	0.66
Trouble breathing	0	4 (5.1)	0.27
Enlarged liver	0	3 (3.8)	0.34
None	0	1 (1.3)	0.59

^a^ Calculated using Chi-square test. ^b^ Multiple response answer. ^c^ Percentages are mutually exclusive.

**Table 3:** Multiple logistic regression model showing factors associated with having family planning influenced by yCRC diagnosis.

	Odds Ratio * (95% Confidence Interval)
Age at yCRC diagnosis **	
40–49 year (ref)	
30–39 year	21.94 (5.59, 86.18)
20–29 year	22.73 (3.53, 146.39)
Cancer site	
Colon (ref)	
Rectum	0.63 (0.20, 1.98)
Both sites	5.06 (0.34, 74.61)
Treatment status **	
Completed treatment (ref)	
In treatment	4.93 (1.29, 18.78)
Had discussion with provider(s)	
No (ref)	
Yes	1.27 (0.43, 3.74)
Unsure	0.33 (0.012, 8.85)
Education	
College or more (ref)	
Less than college	2.03 (0.19, 21.85)

* Odds ratios indicating association with factors and binary outcomes representing participants’ responses to whether their yCRC diagnosis influenced family planning decisions (‘yes’, ‘no’). ** Indicates significant predictors.

**Table 4:** Themes and categories arising from content analysis of responses to open-ended survey questions regarding reproductive and sexual health experiences in relation to yCRC diagnosis (N = 70 participants).

Themes	Categories	Participant-Mentions N (%)	Representative Quote(s)
Emotional impacts of yCRC	(a) Processing diagnosis	4 (5.7)	“My greatest concern, when diagnosed, was will I live through this?” (40–49, never been pregnant) “[The diagnosis] made us realize how precious life is.” (40–49, have been pregnant) “You go into immediate fight mode.” (40–49, have been pregnant)
	(b) Worries and fears	7 (10.0)	“The thought of having a cancer recurrence with a family scares me.” (20–29, never been pregnant) “I am in fear of my future.” (30–39, have been pregnant) “I no longer feel attractive.” (40–49, have been pregnant)
	(c) Coping strategies	3 (4.3)	“I took my first surgeon’s advice and looked at my treatment one step at a time.” (40–49, have been pregnant) “When looked at by each step, [treatment] is so very much easier to deal with.” (40–49, have been pregnant) “I feel my positive attitude helped me a lot.” (40–49, have been pregnant)
Experiences with reproductive healthcare after yCRC diagnosis	(a) Did not have a discussion with healthcare providers	10 (14.3)	“No discussion was ever had … [I] was not given any information, so anything would’ve been good.” (20–29, have been pregnant) “There was really no discussions about reproductive health at the beginning.” (40–49, have been pregnant) “Doctors need to be more proactive with discussing fertility-preservation.” (20–29, never been pregnant)
	(b) Received referral for reproductive healthcare	2 (2.9)	“Referred me to a fertility specialist.” (40–49, never been pregnant) “Refer to gynec re: menopause after treatment ends.” (40–49, have been pregnant)
	(c) Had a discussion with healthcare providers	9 (12.9)	“They only mention that there may be some pain and discharge.” (40–49, have been pregnant) “I was well informed.” (30–39, have been pregnant) “I went through menopause and needed info about this, which my Dr. provided.” (30–39, have been pregnant)
	(a) Online resources	2 (2.9)	“I found online forums to be a huge help in this area.” (40–49, have been pregnant) “[I] have had to turn to online resources.” (40–49, have been pregnant)
Reproductive and family planning considerations with yCRC diagnosis	(a) Pregnancy history	33 (47.2)	“I have two healthy children who were conceived prior to the cancer diagnosis and treatment.” (30–39, have been pregnant) “I had 1 teenage child and one adult child at the time of diagnosis.” (40–49, have been pregnant)
	(a) Impact of yCRC on decisions to have children	19 (27.1)	“After so much surgery and invasion of my body the thought of giving birth or being pregnant makes me feel dread rather than any sense of excitement.” (30–39, never been pregnant) “I was done with ever being pregnant again but the possibility of adopting was on the table. Now, I’m not sure that is a good idea.” (30–39, have been pregnant) “I’ve been unsure if I wanted kids but I think my health has pushed me towards not having children.” (30–39, never been pregnant)
	(b) Role of genetic testing	8 (11.4)	“I was later diagnosed with Lynch syndrome ^a^ and had a full hysterectomy.” (40–49, have been pregnant) “I was able to reflect and confirmed my belief that I would not want children in the future, especially now, given the diagnosis and genetic mutation I am carrying.” (20–29, never been pregnant)
	(c) Fertility	5 (7.1)	“I wish I had been given options to preserve some eggs in case I wanted children in the future.” (20–29, have been pregnant) “In general, there is no solid information about the effects of chemo on fertility. Dr’s said it was an unknown.” (30–39, have been pregnant) “I wish a longer discussion and basic overview of my reproductive health was done.” (30–39, have been pregnant)
Sexual health impacts of yCRC	(a) Vaginal side effects	8 (11.4)	“Vaginal damage was downplayed.” (40–49, have been pregnant) “I also experienced issues with vaginal stenosis. My doctors never informed me about this long term side effect from the treatments.” (20–29, never been pregnant) “Vaginal changes after radiation being severe.” (30–39, have been pregnant)
	(b) Intimacy and intercourse	5 (7.1)	“During and after treatment my interest intimacy was affected and my partner did not understand this, so it would have been good to have some type of counselling as a couple on this topic.” (30–39, have been pregnant) “How difficult sex would be afterwards.” (40–49, have been pregnant) “I did not really digest how this would impact my intimate relationship with my husband.” (40–49, have been pregnant)

^a^ Lynch Syndrome: a type of inherited cancer syndrome which predisposes affected individuals to different types of cancers.

## Data Availability

The data presented in this study are available on request from the corresponding author. The data are not publicly available due to ethics considerations.

## Supplementary Materials

The following supporting information can be downloaded at: https://www.mdpi.com/article/10.3390/curroncol29020042/s1, Figure S1: Overview of survey design.
