# Supplementary figures and images for: Reproductive Health Experiences of Females Diagnosed with Young-Onset Colorectal Cancer: A Multi-Method Cross-Sectional Survey
Source: Curr Oncol. 2022 Jan 21;29(2):465–78. doi: 10.3390/curroncol29020042 (PMC8870126; doi:10.3390/curroncol29020042)

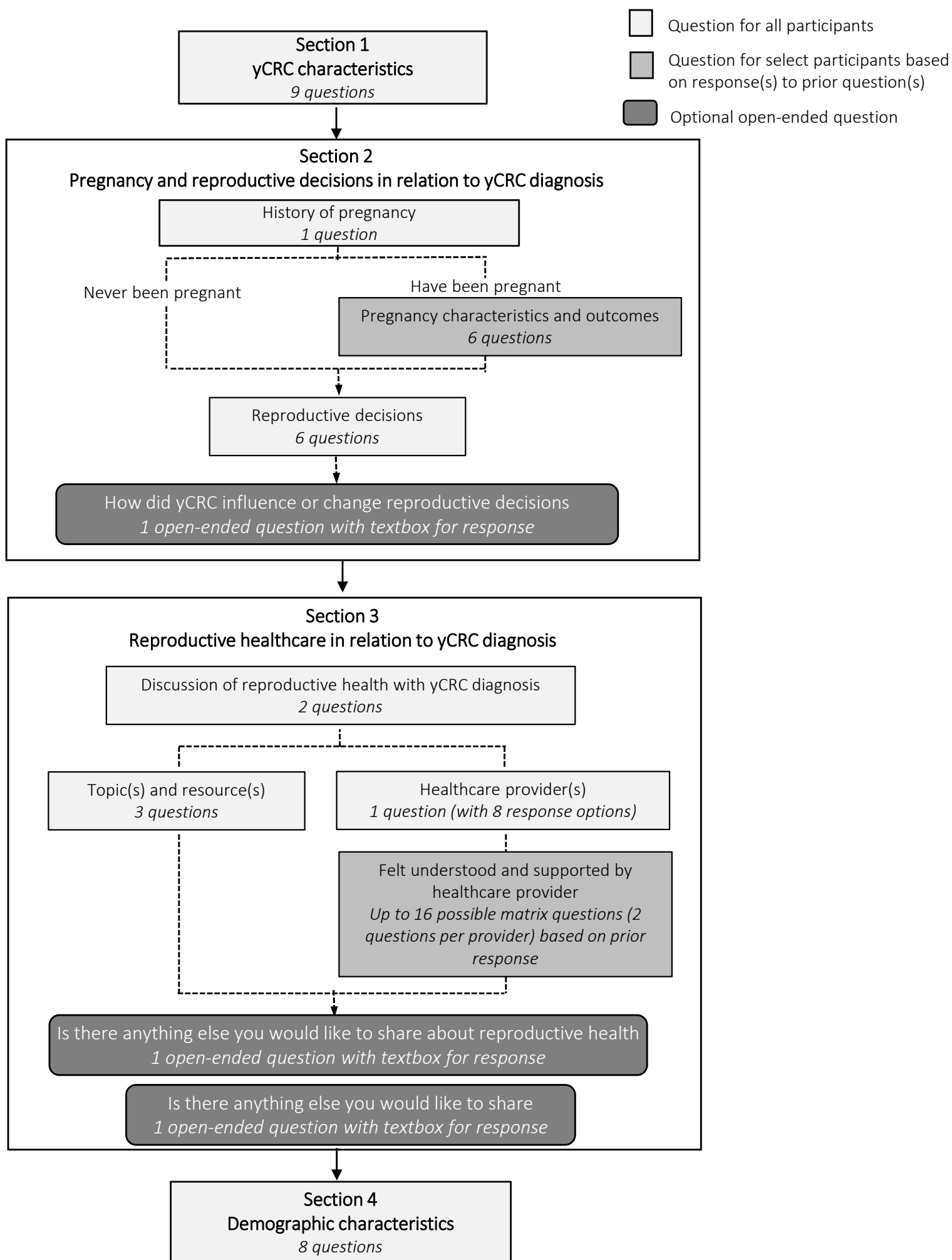

**Supplementary Figure S1.** Overview of survey design

Supplement: Supplementary file 1 [file curroncol-29-00042-s001.zip › curroncol-1515635-supplementary.pdf]
